# Dietary Supplementation of Glutamate Alleviates LPS-Induced Intestinal Injury Associated with m^6^A Modification of SLC10A2 in Weaned Piglets

**DOI:** 10.3390/ani16182854

**Published:** 2026-09-10

**Authors:** Zhending Gan, Jiawei He, Qiyue Jin, Qiuqin Ma, Chuanlong Wang, Xiang Zhong

**Affiliations:** 1 College of Animal Science and Technology, Nanjing Agricultural University, Nanjing 210095, China; gzd273008102@163.com (Z.G.); a13489189020@163.com (J.H.); jinqiyue@stu.njau.edu.cn (Q.J.); 2 Institute of Animal Science, Jiangsu Academy of Agricultural Sciences, Key Laboratory of Crop and Livestock Integration, Ministry of Agriculture and Rural Affairs, Nanjing 210014, China; 20230076@jaas.ac.cn; 3 College of Animal Science and Technology, Yangzhou University, Yangzhou 225000, China; 4 Institute of Animal Nutrition and Health Industry, Anyou Biotechnology, Nanjing Agricultural University, Nanjing 210095, China; 5 Natural Plant and Animal Health Innovation Institute, NJAU-Cohoo Biotechnology, Nanjing Agricultural University, Nanjing 210095, China; 6 State Key Laboratory of Meat Quality Control and Cultured Meat Development, Nanjing Agricultural University, Nanjing 210095, China

**Keywords:** glutamate, weaned piglets, m^6^A modification, SLC10A2/ASBT, bile acid metabolism, FXR

## Abstract

Acute inflammatory challenge can impair intestinal barrier function and bile acid homeostasis in weaned piglets. Glutamate serves as an important energy substrate and functional amino acid for intestinal epithelial cells, yet the epitranscriptomic mechanisms underlying its potential protective effects remain poorly defined. This study investigated whether dietary glutamate supplementation is associated with m^6^A-dependent regulation of SLC10A2 and bile acid signaling in weaned piglets subjected to an acute LPS challenge, with supporting validation in IPEC-J2 cells. LPS challenge (100 μg/kg body weight) impaired ileal morphological structure, activated the TLR4/NF-κB signaling pathway, and aggravated intestinal inflammatory responses in piglets. In contrast, glutamate supplementation was associated with restored ileal mucosal morphology, up-regulated expression of intestinal tight junction proteins, and suppressed TLR4/NF-κB pathway activation. Further metabolomic analysis indicated that LPS stimulation and glutamate intervention altered ileal bile acid metabolism: LPS decreased total bile acid (TBA) content in ileal tissue while increasing TBA accumulation in ileal chyme, whereas glutamate treatment partially reversed this pattern, by elevating ileal tissue TBA and reducing chyme TBA, suggesting a facilitatory effect of glutamate on intestinal bile acid reabsorption. Glutamate supplementation was also associated with increased ileal ASBT (SLC10A2) and FXR (NR1H4) expression. The SLC10A2 transcript exhibited detectable m^6^A modification; glutamate supplementation rescued the LPS-induced downregulation of m^6^A demethylases FTO and ALKBH5, which was associated with reduced global and SLC10A2-specific m^6^A modification. In vitro experiments in IPEC-J2 cells suggested that inhibition of FTO or glutamate dehydrogenase attenuated the effects of glutamate on m^6^A modification and SLC10A2 expression, and that YTHDF2 may contribute to the degradation of hyper-m^6^A-modified SLC10A2 mRNA. Glutamate-derived α-KG may serve as a cofactor supporting demethylase expression and activity after LPS stimulation. Collectively, these findings suggest that glutamate may alleviate LPS-induced intestinal inflammation in weaned piglets through a pathway involving α-KG-dependent maintenance of m^6^A demethylase expression, reduced m^6^A modification of SLC10A2, and preserved ASBT/FXR signaling. This study provides evidence for a nutritional–epitranscriptomic axis that may contribute to intestinal protection under acute inflammatory stress.

## Simple Summary

Weaning stress and acute inflammatory challenges can compromise intestinal barrier function and bile acid homeostasis in piglets. Glutamate is a key energy substrate and functional amino acid for intestinal epithelial cells, but its epitranscriptomic role in intestinal inflammation remains incompletely understood. This study examined whether dietary glutamate supplementation is associated with altered N6-methyladenosine (m^6^A) modification of SLC10A2 and bile acid signaling in an acute LPS challenge model in weaned piglets, with supporting validation in IPEC-J2 cells. LPS challenge disrupted ileal morphology, triggered TLR4/NF-κB-driven inflammation and disturbed bile acid distribution. Glutamate supplementation was associated with restored mucosal integrity, suppressed TLR4/NF-κB signaling, and promoted ileal bile acid reabsorption. Glutamate-derived α-ketoglutarate may support the expression of m^6^A demethylases FTO and ALKBH5, lowering m^6^A modification of SLC10A2 and limiting YTHDF2-mediated SLC10A2 mRNA degradation, which may in turn preserve FXR expression. In vitro assays supported these observations. Collectively, these findings provide evidence for a glutamate–α-KG–m^6^A–SLC10A2–FXR axis that may contribute to intestinal protection under acute inflammatory stress, offering a potential epigenetic target for nutritional intervention in stressed piglets.

## 1. Introduction

Amino acids are fundamental building blocks of proteins. Beyond their essential role in protein synthesis for the growth and development of piglets, amino acids exert crucial regulatory effects on multiple physiological processes, including intestinal homeostasis maintenance and immune function modulation in piglets [1,2]. Glutamate can be catalyzed by glutamate dehydrogenase (GDH) to produce α-ketoglutarate (α-KG). The generated α-KG further participates in the tricarboxylic acid (TCA) cycle to drive adenosine triphosphate (ATP) synthesis, serving as an important energy substrate for the survival and proliferation of intestinal epithelial cells in weaned piglets [3]. With the continuous upgrading of intensive pig breeding technologies, the growth rate and muscle proliferation efficiency of modern piglets have been markedly improved, which substantially elevates systemic energy and nutrient requirements [4]. Nevertheless, early-weaned piglets exhibit inherent physiological imperfections, characterized by immature intestinal structure and function. Combined with the stress induced by the transition from breast milk to solid feed, the digestive, absorptive, and metabolic capacities of amino acids are significantly impaired, leading to insufficient endogenous supply of functional amino acids represented by glutamate [5]. Besides this, glutamate is one of the core functional amino acids for maintaining immune cell metabolism and the physiological functions of immune cells. The activation of glutamate transporters in immune cells enhances cellular glutamate uptake, whereas glutamate deficiency directly suppresses the activation of pivotal immune cells, such as macrophages and mast cells [6,7]. Previous studies have indicated that supplementing appropriate amino acids in feed can effectively modulate intestinal immune function and alleviate weaning stress-induced intestinal injury in piglets [8,9]. However, the specific molecular mechanism by which amino acids regulate piglet immune cell function remains incompletely understood, particularly with regard to regulation at the epigenetic level.

N6-methyladenosine (m^6^A) is the most abundant and functionally pivotal epitranscriptomic modification on eukaryotic mRNAs. Through a dynamically reversible methylation and demethylation cycle, m^6^A modification precisely modulates mRNA splicing, stability, and translation efficiency, thereby participating extensively in diverse physiological processes including metabolism, immune response, and growth [10,11]. Emerging evidence has revealed that m^6^A epitranscriptomic modification precisely regulates the inflammatory response of macrophages by modulating the alternative splicing patterns and translation efficiency of immune-related genes [12,13,14]. In piglet research, m^6^A modification is closely correlated with piglet health status. Aberrant fluctuations in mRNA m^6^A methylation levels can impair the expression stability of target genes, thereby restricting the improvement of immune function and growth performance in piglets. Stress exposure can disrupt the homeostasis of m^6^A modification in core metabolic and immune organs (e.g., liver and intestine) of piglets, leading to obvious dysregulation of m^6^A modification levels. Notably, dietary supplementation with functional substances effectively reverses stress-induced m^6^A modification disorders, improves antioxidant capacity and immune status, and enhances average daily gain in piglets [15,16,17]. Collectively, these findings demonstrate that m^6^A epitranscriptomic modification plays an indispensable role in regulating stress response, immune function, and growth and development in piglets.

Consistent with the regulatory patterns of DNA and histone methylation, the biological activities of the m^6^A demethylases FTO and ALKBH5 are strictly dependent on two critical cofactors, α-KG and Fe^2+^. Intracellular α-KG abundance may influence the homeostasis of m^6^A modification, thereby modulating the functional activity of immune cells [18,19]. In typical pro-inflammatory immune cells such as macrophages, the isocitrate dehydrogenase-mediated conversion of isocitrate to α-KG can be impeded [20]. Under this condition, the glutamate metabolic pathway may serve as a pivotal compensatory mechanism to replenish the intracellular α-KG pool and maintain normal cellular physiological functions. The GDH-catalyzed conversion of glutamate to α-KG represents a potential metabolic node for sustaining m^6^A methylation homeostasis and normal immune function in immune cells. Adequate supplies of glutamate and α-KG may be important for stabilizing intracellular m^6^A modification levels and preserving host immune homeostasis [21,22]. In weaned piglets, weaning stress may induce glutamate metabolic imbalance and impair endogenous glutamate and α-KG synthesis, thereby inhibiting the enzymatic activities of FTO and ALKBH5, and disrupting the dynamic balance of m^6^A modification in immune cells. However, whether glutamate supplementation can regulate intestinal bile acid transport through m^6^A-dependent mechanisms in the context of acute inflammatory challenge remains unknown. We therefore hypothesized that dietary glutamate supplementation, through conversion to α-KG, may support m^6^A demethylase expression, reduce m^6^A modification of the bile acid transporter SLC10A2, and thereby preserve ASBT expression and FXR signaling to attenuate LPS-induced intestinal inflammation in weaned piglets. This hypothesis was tested using dietary challenge design in vivo and pharmacological inhibition in IPEC-J2 cells in vitro.

## 2. Materials and Methods

### 2.1. Animals and Experimental Design

One hundred piglets (50 male and 50 female) weaned at 21 ± 2 d of age were obtained from a commercial farm and housed in an environmentally controlled room (25.0 ± 0.5 °C) with *ad libitum* access to feed and water. After a 3 d acclimation period, piglets were blocked by sex and body weight and randomly assigned to one of two dietary treatments (n = 5 pens per treatment, 10 piglets per pen): a control group (CON) receiving a basal diet supplemented with 0.6% L-alanine (Psaitong Biotechnology, Beijing, China, A10001, purity ≥ 99%) as an isonitrogenous control, and a glutamate group (GLU) receiving the basal diet supplemented with 1% L-glutamate (Psaitong Biotechnology, Beijing, China, G10016, purity ≥ 99%). The alanine supplement was included to balance the additional nitrogen contributed by 1% glutamate; both diets were formulated to be equivalent in nitrogen, energy, and major nutrients, and met the NRC (2012) nutrient requirements for weaned piglets. The complete dietary composition is provided in Table S1. The experimental unit for the dietary treatment was the pen (n = 5 per dietary group). Sex was balanced within each pen (5 males and 5 females per pen where possible) and across dietary treatments. The sample size of five pens per dietary treatment was selected based on previous piglet nutrition studies from our laboratory and others using similar endpoints [8,9,15], which consistently detected biologically meaningful differences with n = 4–6 pens per group. All piglets were individually identified by ear tag and monitored daily for general health, feed intake, and any signs of illness throughout the 28 d feeding period.

### 2.2. LPS Challenge and Sample Collection

At 49 d of postnatal age (28 d after weaning), four piglets per pen (two males and two females, selected on the basis of average body weight within the pen) were chosen for the LPS challenge phase, yielding 20 piglets per dietary treatment (5 pens × 4 piglets). Within each dietary treatment, the 20 selected piglets were randomly assigned to either saline or LPS challenge (10 piglets per challenge subgroup per dietary treatment), with the constraint that each pen contributed two piglets to the saline subgroup and two to the LPS subgroup. This resulted in a 2 × 2 factorial arrangement with four final groups: CON (basal diet + 0.6% alanine + saline injection), LPS (basal diet + 0.6% alanine + LPS injection), GLU (basal diet + 1% glutamate + saline injection), and GLS (basal diet + 1% glutamate + LPS injection). For the dietary effect, the experimental unit was the pen (n = 5); for analyses at the individual level, pig was treated as the experimental unit with pen included as a random effect where appropriate. LPS (*E. coli* serotype 055: B5; L2880, Sigma Aldrich, Inc., St. Louis, MO, USA) was dissolved in sterile 0.9% saline and administered by intraperitoneal injection at a dose of 100 μg/kg body weight (injection volume: 1 mL/kg body weight). The dose was selected based on previous studies [8,9]. Control piglets received an equivalent volume of sterile saline by the same route. After injection, piglets were continuously monitored for behavioral signs of distress (lethargy, recumbency, vocalization, respiratory distress), and humane endpoints were predefined: any piglet exhibiting severe respiratory distress, unresponsiveness, or inability to stand would be immediately euthanized. No piglets reached these endpoints during the 4 h observation period. At 4 h after LPS or saline administration, piglets were electrically stunned and exsanguinated. Blood samples were collected from the anterior vena cava. The entire small intestine, from the pyloric sphincter to the ileocecal valve, was rapidly removed and divided into duodenum, jejunum, and ileum. Ileal segments were gently flushed with ice-cold physiological saline to remove luminal contents. Approximately 1 cm sections from the mid-jejunum and mid-ileum were fixed in 4% paraformaldehyde for histological analysis. Ileal mucosa was scraped from the mid-ileum using a glass microscope slide, snap-frozen in liquid nitrogen, and stored at −80 °C for subsequent molecular and biochemical analyses. Ileal chyme was also collected and stored at −80 °C for bile acid quantification.

### 2.3. Cell Culture

IPEC-J2 cells (porcine intestinal epithelial cell line) were cultured in Dulbecco’s modified Eagle’s medium (DMEM, Thermo Fisher Scientific, Waltham, MA, USA, 11965092) supplemented with 10% fetal bovine serum (Wisent Biotechnology Inc., Ottawa, ON, Canada, 086-150) and 1% penicillin–streptomycin (Thermo Fisher Scientific, Waltham, MA, USA 15140148**)** (complete DMEM), and maintained in a 5% CO_2_ incubator at 37 °C. For LPS challenge experiments, cells at 70–80% confluence were treated with LPS (*E. coli* serotype O111:B4, Sigma-Aldrich St. Louis, MO, USA) at a final concentration of 1 μg/mL for 24 h, with or without L-glutamate supplementation at 5 mM. For inhibitor experiments, cells were pretreated for 2 h with one of the following inhibitors before LPS and/or glutamate exposure: FB23-2 (10 μM, FTO inhibitor), DC-Y13-27 (5 μM, YTHDF2 inhibitor), or R162 (10 μM, glutamate dehydrogenase inhibitor). All inhibitors were dissolved in dimethyl sulfoxide (DMSO), and control cells received an equivalent concentration of DMSO (≤0.1%). Cells were harvested at 24 h after LPS exposure for RNA, protein, and metabolite analyses.

### 2.4. Intestinal Morphology

The intestinal segments fixed in 4% paraformaldehyde were dried using a graded series of xylene and ethanol, and embedded in paraffin. The samples (5 μm) were then deparaffinized using xylene and rehydrated with graded dilutions of ethanol. The slides were stained with hematoxylin and eosin. Six slides for each tissue were prepared, and the images were acquired using an optical binocular microscope with a digital camera (Nikon ECLIPSE 80i, Tokyo, Japan).

### 2.5. Western Blotting

The total protein concentration was determined using bicinchoninic acid (BCA) kit (Beyotime Biotechnology, Shanghai, China, P0011). Equal amounts of protein were used for the following experiment. The proteins were transferred onto a PVDF membrane (BioRad, Hercules, CA, USA) after SDS-PAGE electrophoretic separation. The membranes were blocked with BSA, and incubated with primary antibodies and secondary antibodies sequentially. Finally, the developed blots on the membranes were observed and analyzed. The primary antibodies are listed in Table S2.

### 2.6. Real-Time Quantitative PCR (RT-qPCR)

Total RNA was extracted by VeZol reagent (Vazyme Biotech Co., Ltd., Nanjing, China, R411-01), and reversed by Primer Script TM RT reagent Kit (Vazyme Biotech Co., Ltd., Nanjing, China, R333-01) according to the manufacturer’s instructions. RT-qPCR was conducted with SYBR Green on the Quant Studio 6 Real-time PCR system (Thermo Fisher Scientific, Waltham, MA, USA). The samples were individually normalized using β-actin. The primers are listed in Table S3.

### 2.7. Metabolomics Analysis

Non-targeted metabolomics analysis was performed on ileal mucosa samples (n = 6 per group, selected from CON, LPS, and GLS groups) by LC-Bio Technology Co., Ltd. (Hangzhou, China). Briefly, frozen ileal mucosa (~50 mg) was homogenized in 500 μL of prechilled methanol–water (4:1, *v*/*v*) containing 0.1% formic acid, followed by vortexing and sonication on ice for 10 min. The homogenate was centrifuged at 14,000× *g* for 15 min at 4 °C, and the supernatant was collected and vacuum-dried. The dried residue was reconstituted in 100 μL of acetonitrile–water (1:1, *v*/*v*), vortexed, and centrifuged at 14,000× *g* for 10 min at 4 °C. The supernatant was transferred to an LC vial for analysis. Chromatographic separation was performed on an ultra-high-performance liquid chromatography (UHPLC) system (Vanquish, Thermo Fisher Scientific, Waltham, MA, USA) equipped with a HILIC column (2.1 × 100 mm, 1.7 μm; Waters). The mobile phase consisted of (A) 25 mM ammonium acetate and 25 mM ammonium hydroxide in water and (B) acetonitrile, with a gradient elution program of 0–0.5 min, 95% B; 0.5–7 min, 95–65% B; 7–8 min, 65–40% B; 8–9 min, 40% B; 9–9.1 min, 40–95% B; and 9.1–12 min, 95% B. The flow rate was 0.3 mL/min, and the injection volume was 2 μL. Mass spectrometry was performed on a Q Exactive HF-X mass spectrometer (Thermo Fisher Scientific, Waltham, MA, USA) in both positive and negative electrospray ionization modes. Raw data were processed using Compound Discoverer 3.1 (Thermo Fisher Scientific, Waltham, MA, USA) for peak alignment, retention time correction, and metabolite identification against the HMDB, Metlin, and mzCloud databases. Differential metabolites were identified using a threshold of variable importance in projection (VIP) > 1, fold change > 1.2 or <0.83, and *p* < 0.05 (Student’s *t*-test).

### 2.8. Cytokine Detection

Cytokine levels (IL-1β and TNF-α) in piglet ileal tissue were measured using commercial enzyme-linked immune response (ELISA) kits (CUSABIO technology, Wuhan, China, CSB-E06782p, B-E16980p-IS, https://www.cusabio.com/) according to the manufacturer’s instructions.

### 2.9. Total Bile Acid Detection

The bile acid content in ileum and ileal content were measured by commercial reagent kit (Jiancheng Biotechnology, Nanjing, China, E003-2-1) according to the instructions provided by the manufacturer.

### 2.10. M^6^A Content Detection

The m^6^A content in the ileum and cell was detected using the EpiQuikTM m^6^A RNA Methylation Quantification Kit (Epigentek, New York, NY, USA, P-9005). Briefly, 200 ng RNA was mixed with 80 μL binding solution, added to the 96-well plate provided by the kit, and incubated at 37 °C for 90 min. After incubation, the solution was discarded. Subsequently, the samples were incubated with capture antibodies for 60 min, detection antibodies for 30 min, and enhancement solution for 30 min. After each incubation, the solution weas removed and the well plate was washed with washing buffer. Color reagent was added to each well and incubated in the dark for 8 min. Finally, termination solution was added to stop the reaction and the absorbance was measured at a wavelength of 450 nm (Thermo Fisher, Multiskan Go, Waltham, CA, USA).

### 2.11. MeRIP-qPCR

The relative abundance of SLC10A2 mRNA in m^6^A antibody IP samples and input samples was assessed by qRT-PCR. Total RNA was isolated from mucosa with VeZol reagent (Vazyme). The immunoprecipitation assay was performed by using a EpiMark N6 methyladenine enrichment kit (New England Biolabs, Boston, MA, USA, E1610S) as per previously studies [23,24]. The specific steps were as follows: Firstly, protein G magnetic beads (New England Biolabs, MA, USA, S1430,) were incubated with m^6^A antibody in reaction buffer (150 mM NaCl, 10 mM Tris HCl, and 0.1% NP-40) at 4 °C for 30 min. Subsequently, the mRNA and protein G-m^6^A antibody complex was incubated in IP buffer (750 mM NaCl, 50 mM Tris HCl, and 0.5% NP-40) at 4 ° C for 1 h. To remove non-specific binding molecules, the magnetic beads were washed sequentially with three different buffers: reaction buffer, low-salt buffer (50 mM NaCl, 10 mM Tris HCl, 0.1% NP-40), and high-salt buffer (500 mM NaCl, 10 mM Tris HCl, 0.1% NP-40). Magnetic beads were resuspended in 100 μ L RLT buffer (Cat: 1015762, QIAGEN, Düsseldorf, Germany) and incubated for 1 min to release RNA. Finally, RNA purification was performed using an RNA purification and concentration kit (Zymo Research, Orange County, CA, USA, R1017), and then detected by qRT-PCR. The primers are listed in Table S4.

### 2.12. Glutamate and α-KG Detection

Glutamate and α-KG in ileal and lysed cells were analyzed by Liquid Chromatography–Mass Spectrometry (LC-MS) (Vanquish, Thermo Fisher, Waltham, MA, USA) [25]. The 800 μL methanol–acetonitrile (1:1) was mixed with 200 μL 10% ileum homogenate solution or cell extract and centrifuged at 14,000 rpm for 15 min at 4 °C. The supernatants were vacuum-dried at 60 °C for 2 h and further dried with nitrogen to ensure complete dryness. The dry residues were dissolved in 200 μL methanol–water (1:1) and sonicated on ice. The supernatants were filtered through a 0.22 μm membrane filter and stored at −20 °C before LC-MS analysis.

### 2.13. Statistical Analysis

Data in the current study were analyzed statistically using Graphpad Prism 8.0 software and presented as means ± SD. The animal experiment followed a 2 × 2 factorial arrangement (dietary glutamate supplementation × LPS challenge), and the primary statistical model was a one-way analysis of variance (ANOVA) with dietary treatment, LPS challenge, and their interaction as fixed effects. For two-group comparison, data were analyzed using two-tailed Student’s *t*-test if the data were in Gaussian distribution and had equal variance, or by Mann–Whitney U test if the data were not normally distributed. *p* < 0.05 was defined as a significant difference, and data in figures are presented as * *p* < 0.05, ** *p* < 0.01, *** *p* < 0.001 and **** *p* < 0.0001.

## 3. Results

### 3.1. Dietary Supplementation of Glutamate Improves Ileal Morphology and Alleviates Ileal Inflammation in LPS-Challenged Piglets

**Figure 1:**
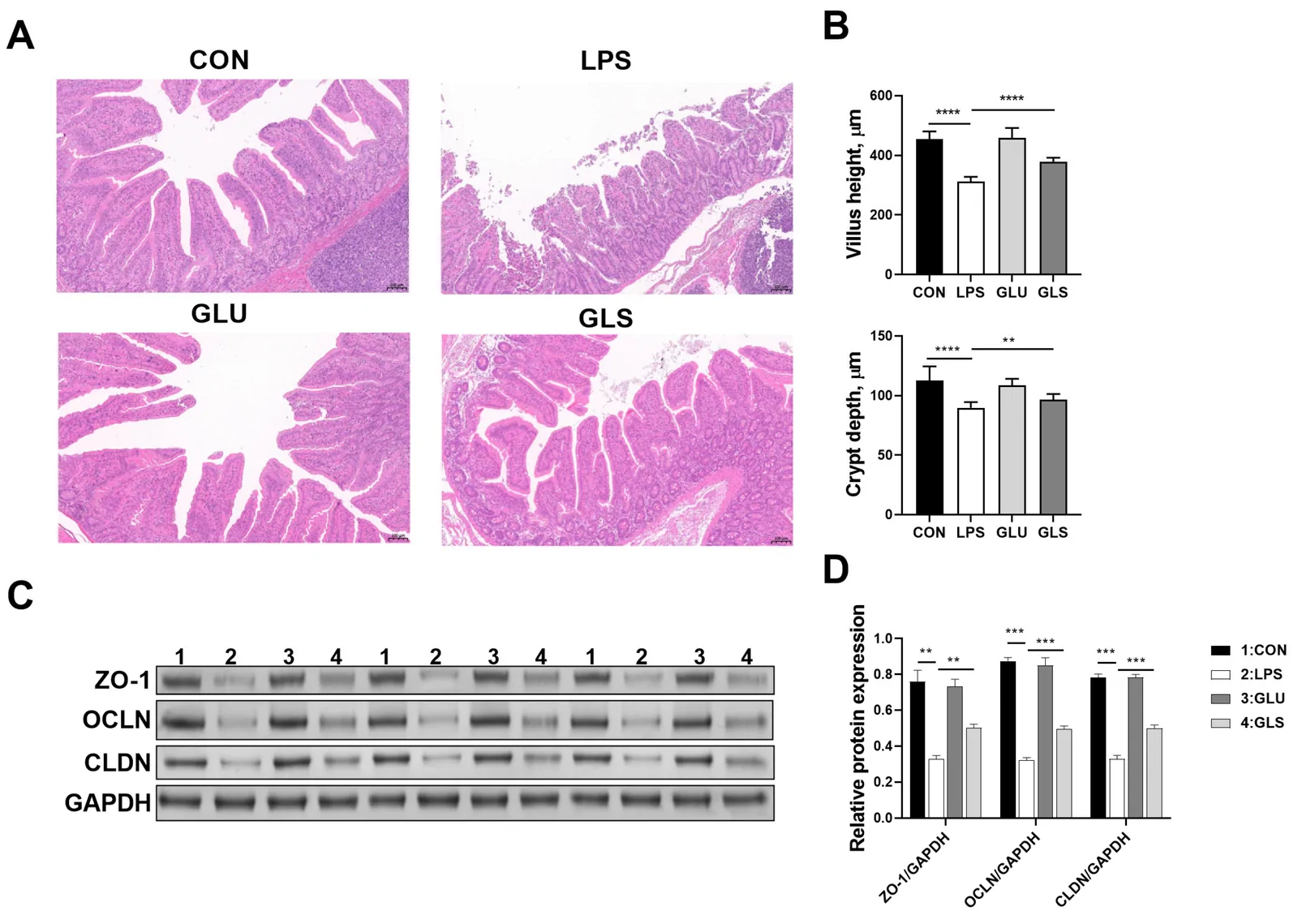
Effect of dietary glutamate supplementation on the morphology of the ileal mucosa in piglets. (**A**) H&E staining of piglet ileal tissue in the control (CON), LPS challenge (LPS), glutamate supplementation (GLU) and LPS challenge with glutamate supplementation (GLS) groups (n = 3), scale bar, 100 μm. (**B**) Villus height (μm) and crypt depth from (**A**). (**C**,**D**) Protein abundance of ZO-1, OCLN and CLDN. (**A**–**D**), n = 3 replicates. Data were analyzed using two-tailed Student’s *t*-test, and represented as means ± SD. ** *p* < 0.01, *** *p* < 0.001 and **** *p* < 0.0001. ZO-1, zonula occludens-1; OCLN, occludin; CLDN, claudin; H&E, hematoxylin and eosin.

To explore the protective effects of glutamate on intestinal injury induced by lipopolysaccharide (LPS) challenge, intestinal morphological characteristics and tight junction protein expression in the ileum were determined, given that the ileum is the primary intestinal segment affected by LPS stimulation. The results showed that LPS challenge markedly decreased ileal villus height, increased crypt depth (Figure 1A,B), and down-regulated the protein levels of intestinal tight junction markers, including zonula occludens-1 (ZO-1), occludin (OCLN), and claudin (CLDN) (Figure 1C,D). Notably, dietary glutamate supplementation effectively reversed LPS-induced intestinal morphological damage, as evidenced by increased villus height and reduced crypt depth (Figure 1A,B). Moreover, glutamate supplementation restored the decreased expression of tight junction proteins in LPS-challenged piglets (Figure 1C,D). In contrast, dietary glutamate intervention exerted no significant effects on ileal morphological structure and tight junction protein expression in unchallenged control piglets.

**Figure 2:**
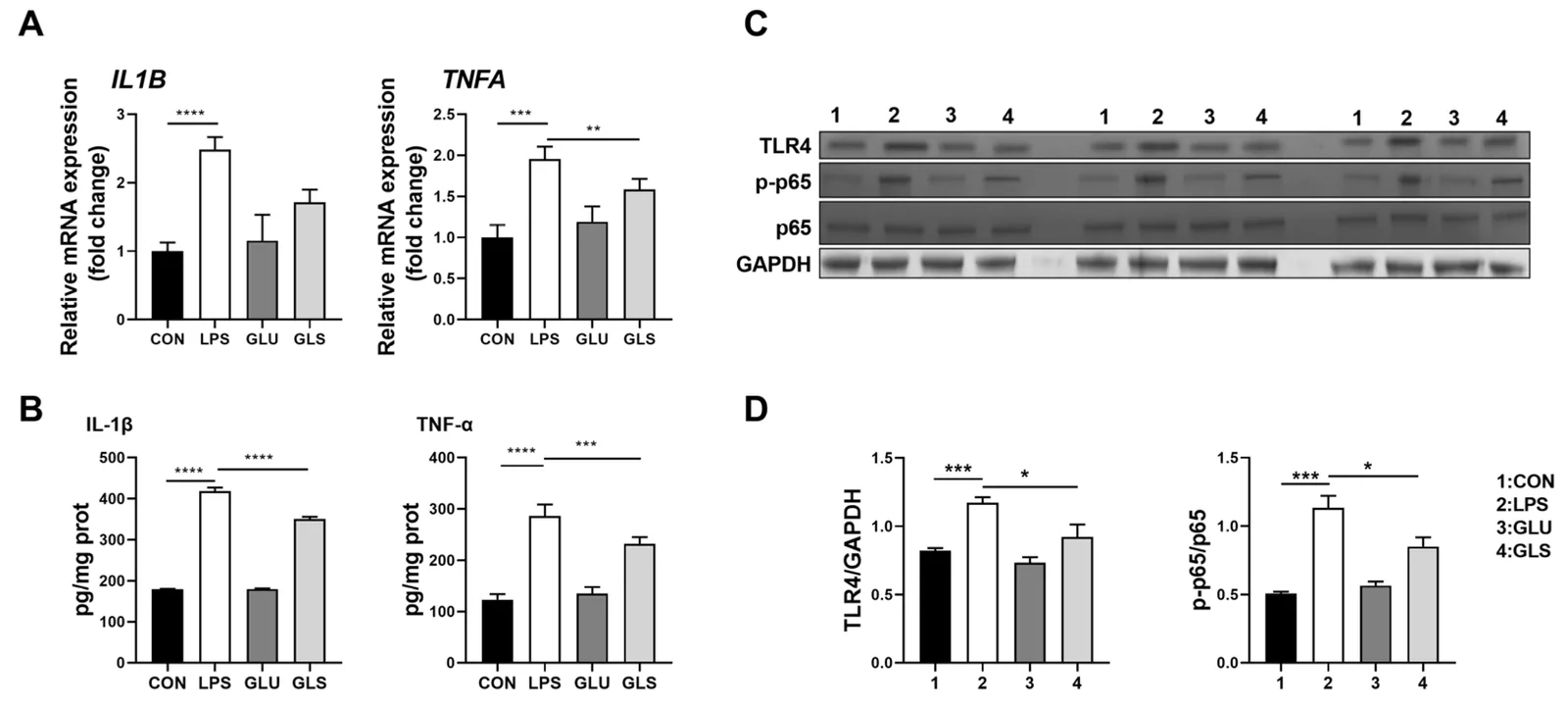
Dietary supplementation of glutamate influences ileal inflammation via suppression of the TLR4/NF-κB pathway in piglets. (**A**) Relative mRNA expression of Il1B and TNFA in the control (CON), LPS challenge (LPS), glutamate supplementation (GLU) and LPS challenge with glutamate supplementation (GLS) groups (n = 4 replicates). (**B**) IL-1β and TNF-α levels in the ileal tissue (n = 4 replicates). (**C**,**D**) Protein abundance of TLR4, p-p65 and p65 in piglet ileal tissue (n = 3 replicates). Data were analyzed using two-tailed Student’s *t*-test, and represented as means ± SD. * *p* < 0.05, ** *p* < 0.01, *** *p* < 0.001 and **** *p* < 0.0001. IL1B/IL-1β, interferon-1 beta; TNFA/TNF-α, tumor necrosis factor alpha; TLR4, toll-like receptor 4.

LPS stimulation robustly activates pro-inflammatory signaling cascades and promotes the secretion of pro-inflammatory cytokines, ultimately leading to intestinal mucosal injury. Accordingly, we further determined the inflammatory status in the ileum of piglets. The results showed that LPS challenge significantly up-regulated the mRNA expression of *IL1B* and *TNFA* as well as the protein levels of IL-1β and TNF-α in the ileum, whereas dietary glutamate supplementation effectively reversed these elevations (Figure 2A,B). In addition, glutamate supplementation inhibited the LPS-induced up-regulation of TLR4 expression and reduced the phosphorylation level of p65 (Figure 2C,D). Collectively, these findings indicate that glutamate alleviates LPS-induced ileal inflammatory responses and subsequent morphological damage in weaned piglets.

### 3.2. Glutamate Influences Bile Acid Metabolism by Improving Bile Acid Reabsorption in Weaned Piglets upon LPS Challenge

**Figure 3:**
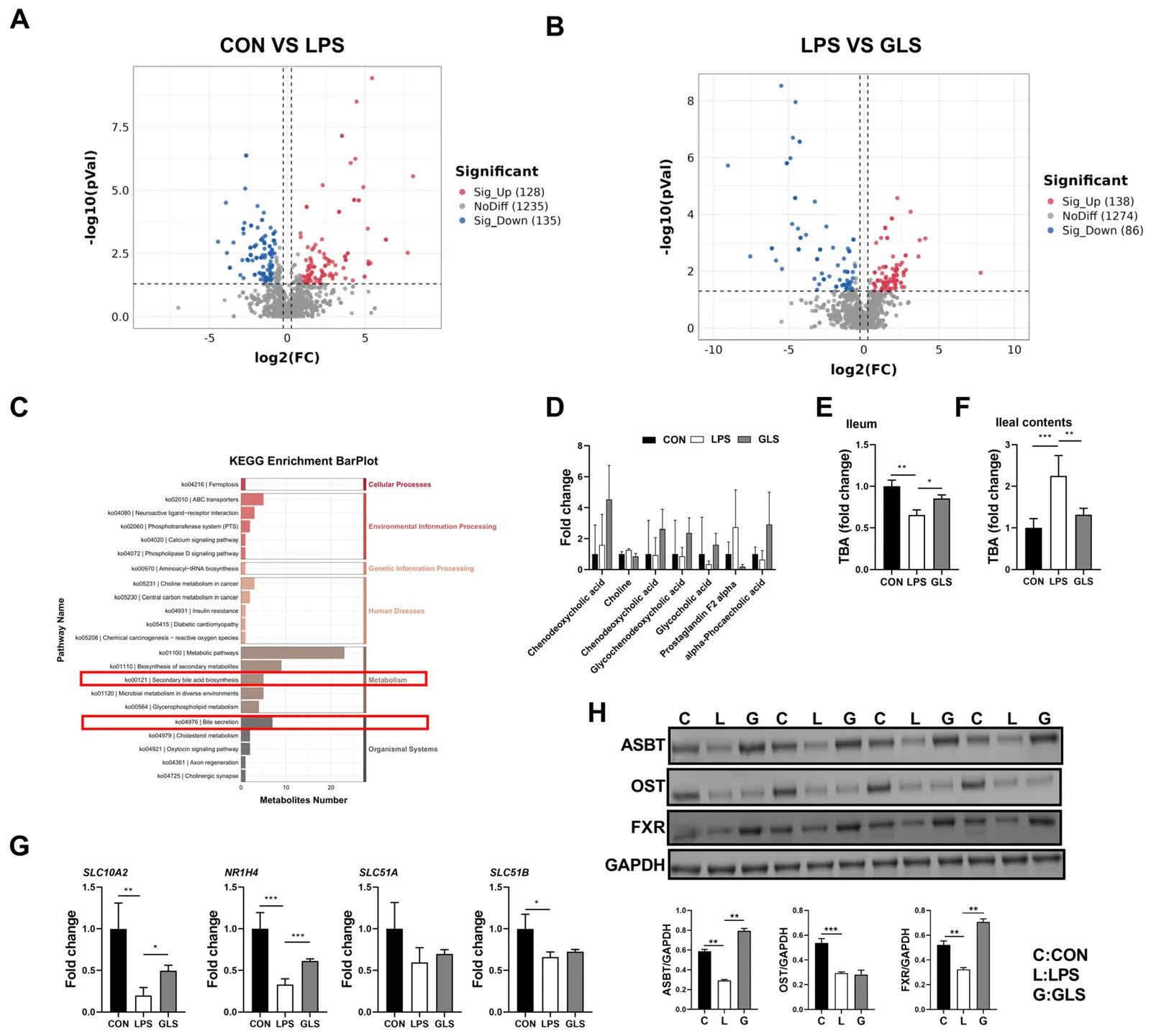
LPS stimulation and glutamate intervention altered ileal metabolism in piglets. (**A**,**B**) Volcano plot of differential metabolites of piglet ileal tissue in the control (CON) VS LPS challenge (LPS) group (**A**) and LPS VS LPS challenge with glutamate supplementation (GLS) group (n = 6 replicates). (**C**) Kyoto Encyclopedia of Genes and Genomes (KEGG) enrichment analysis of differential metabolites of the ileum. (**D**) Relative abundance of differential metabolites from secondary bile acid biosynthesis and bile secretion pathway. (**E**,**F**) Relative abundance of total bile acid in the ileum and ileal content of piglets (n = 4 replicates). (**G**) Relative mRNA expression level of bile acid reabsorption (n = 4 replicates). (**H**) Protein abundance of ASBT, OST and FXR in piglets (n = 4 replicates). Data were analyzed using two-tailed Student’s *t*-test or Mann–Whitney U test (G- *SLC10A2* and *SLC51A*), and represented as means ± SD. * *p* < 0.05, ** *p* < 0.01 and *** *p* < 0.001. ASBT, apical sodium-dependent bile acid transporter; OST, organic solute transporter; FXR, farnesoid X receptor; TBA, total bile acid; KEGG, Kyoto Encyclopedia of Genes and Genomes.

To explore how glutamate alleviates ileal inflammation in LPS-challenged piglets, especially metabolic ways, we conducted metabolomics analysis on the ileum of piglets in the control group (CON), LPS stimulation group (LPS), and LPS stimulation plus glutamate intervention group (GLS). Given that the glutamate supplementation group (GLU) showed no significant effects at the intestinal morphology and inflammatory levels, we did not further test the GLU group in the following experiments. We identified 128 up-regulated differential metabolites (DMs) and 135 down-regulated DMs in the CON group compared with the LPS group (Figure 3A), and 138 up-regulated DMs and 86 down-regulated DMs in the LPS group compared with the GLS group (Figure 3B). Of note, Kyoto Encyclopedia of Genes and Genomes (KEGG) analysis demonstrated that DMs are significantly enriched in pathways related to bile acid (BA) metabolism in both metabolism and organismal system pathways (Figure 3C). These BA-related metabolites showed significant changes after LPS stimulation and glutamate intervention. We also measured the total bile acids (TBAs) in the ileal tissue and ileal chyme; the results showed that LPS challenge significantly decreased TBA in the ileum while increasing TBA in the ileal contents, which indicated that LPS challenge impaired the reabsorption of BAs in the ileum, while glutamate supplementation effectively restored TBA reabsorption (Figure 3E,F).

Next, we investigated how glutamate regulates ileal BA reabsorption. LPS reduced the expression of SLC10A2 (with coding ASBT, intake BAs in the intestine) and SLC51A (with coding OST, transport BAs from intestinal cells to portal vein), while glutamate rescued SLC10A2/ASBT expression in ileal tissue (Figure 3G,H). In addition, glutamate also replenished the decrease in *NR1H4* (with coding FXR, perceive BA levels of intestinal cells) (Figure 3G,H). FXR plays a bridging role between intestinal cell BAs and inflammation, and its elevation can directly inhibit inflammatory pathways like TLR4 and NF-κB to suppresses intestinal inflammatory responses [26,27]. Altogether, glutamate restored bile acid reabsorption, alleviating inflammatory responses via the ileal ASBT-FXR pathway.

### 3.3. Glutamate Supplementation Affects m^6^A Modification and Demethylase Expression Levels in the Ileum of LPS-Challenged Piglets

**Figure 4:**
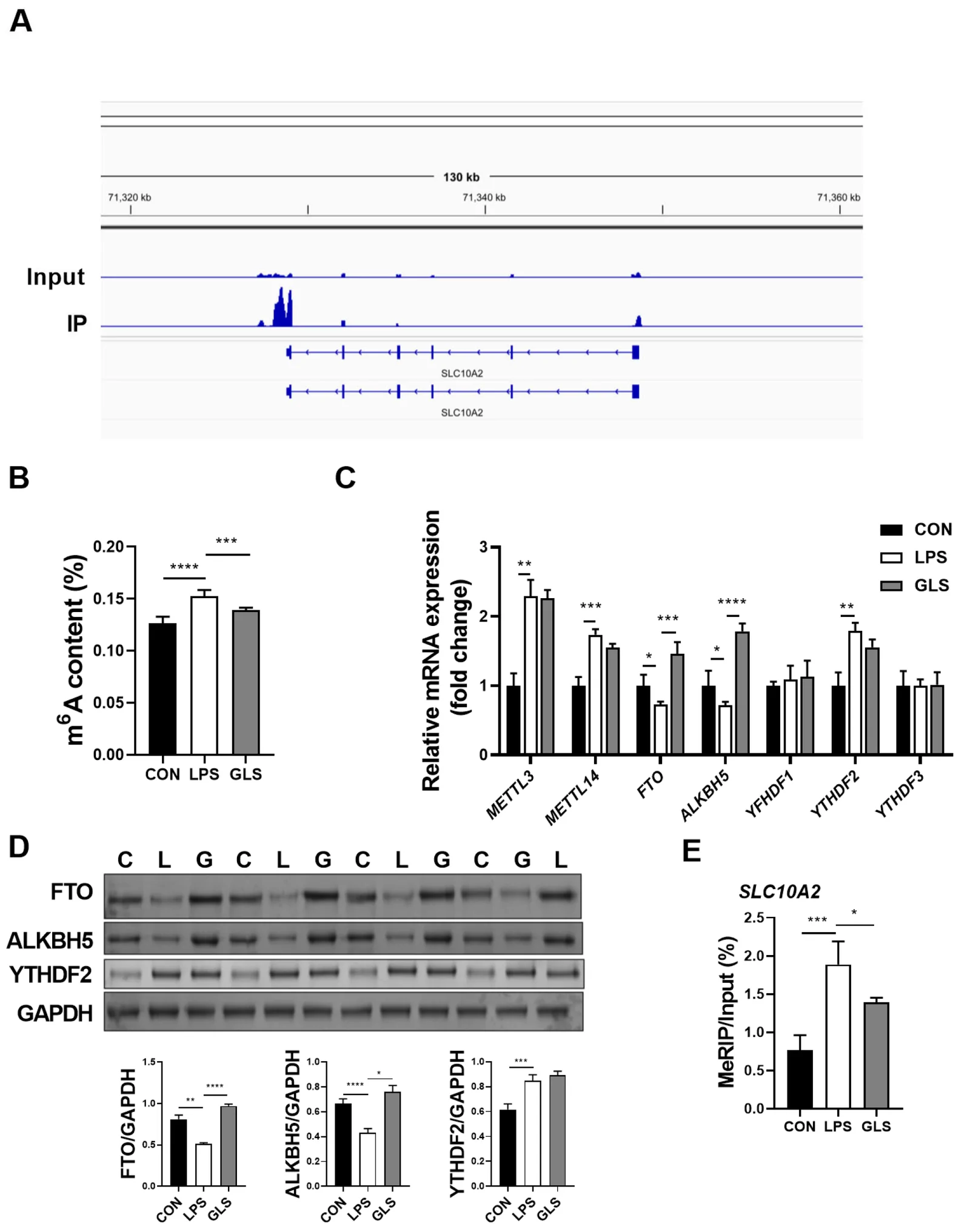
Effects of glutamate supplementation on m^6^A modification of *SLC10A2* via upregulation of m^6^A demethylase expression. (**A**) Distribution of m^6^A of *SLC10A2* in the ileum of weaned piglets. (**B**) m^6^A content of piglet ileum from the control (CON), LPS challenge (LPS), and LPS challenge with glutamate supplementation (GLS) groups (n = 6 replicates). (**C**) Relative mRNA expression levels of genes related to m^6^A methylation (n = 4 replicates). (**D**) Protein abundance of FTO, ALKBH5 and YTHDF2 of piglet ileum (n = 4 replicates). (**E**) MeRIP-qPCR analyzed the m^6^A abundance on SLC10A2 (n = 4 replicates). Data were analyzed using two-tailed Student’s *t*-test or Mann–Whitney U test (D-ALKBH5/GAPDH), and represented as means ± SD. * *p* < 0.05, ** *p* < 0.01, *** *p* < 0.001 and **** *p* < 0.0001. METTL, methyl transferase-like; FTO, fat mass and obesity-associated protein; ALKBH5, AlkB homolog 5; YTHDF, YTH N6-methyladenosine RNA-binding protein; MeRIP-qPCR, methylated RNA immunoprecipitation quantitative PCR.

RNA demethylases including fat mass and obesity-associated protein (FTO) and AlkB homolog 5 (ALKBH5) require α-KG as a substrate; thus, we speculated that dietary supplementation of glutamate in LPS-challenged piglets alters m^6^A demethylase expression by altering m^6^A modification of BA metabolism proteins. The results from the m^6^A-RIP-sequencing database show that the SLC10A2 gene in the ileum of piglets undergoes m^6^A modification (Figure 4A). As expected, the addition of glutamate reduced hyper-m^6^A modification levels in the ileum (Figure 4B). LPS stimulation increased the expression of m^6^A methyltransferases *METTL3* and *METTL14*, as well as elevating the reader *YTHDF2* expression, and decreased the expression of demethylases *FTO* and *ALKBH5*. The addition of glutamate restored the expression of *FTO* and *ALKBH5* (Figure 4C). The results of protein expression analyses further confirm the replenishment effect of glutamate on m^6^A demethylase (Figure 4D). Of note, glutamate reduces the levels of m^6^A modification in the mRNA of SLC10A2 (Figure 4E). Overall, these data imply that glutamate supplementation can decrease m^6^A modification in the LPS-challenged piglet ileum.

### 3.4. Glutamate Upregulation of SLC10A2/ASBT Expression in LPS-Stimulated IPEC-J2 Cells by m^6^A Demethylase and YTHDF2

**Figure 5:**
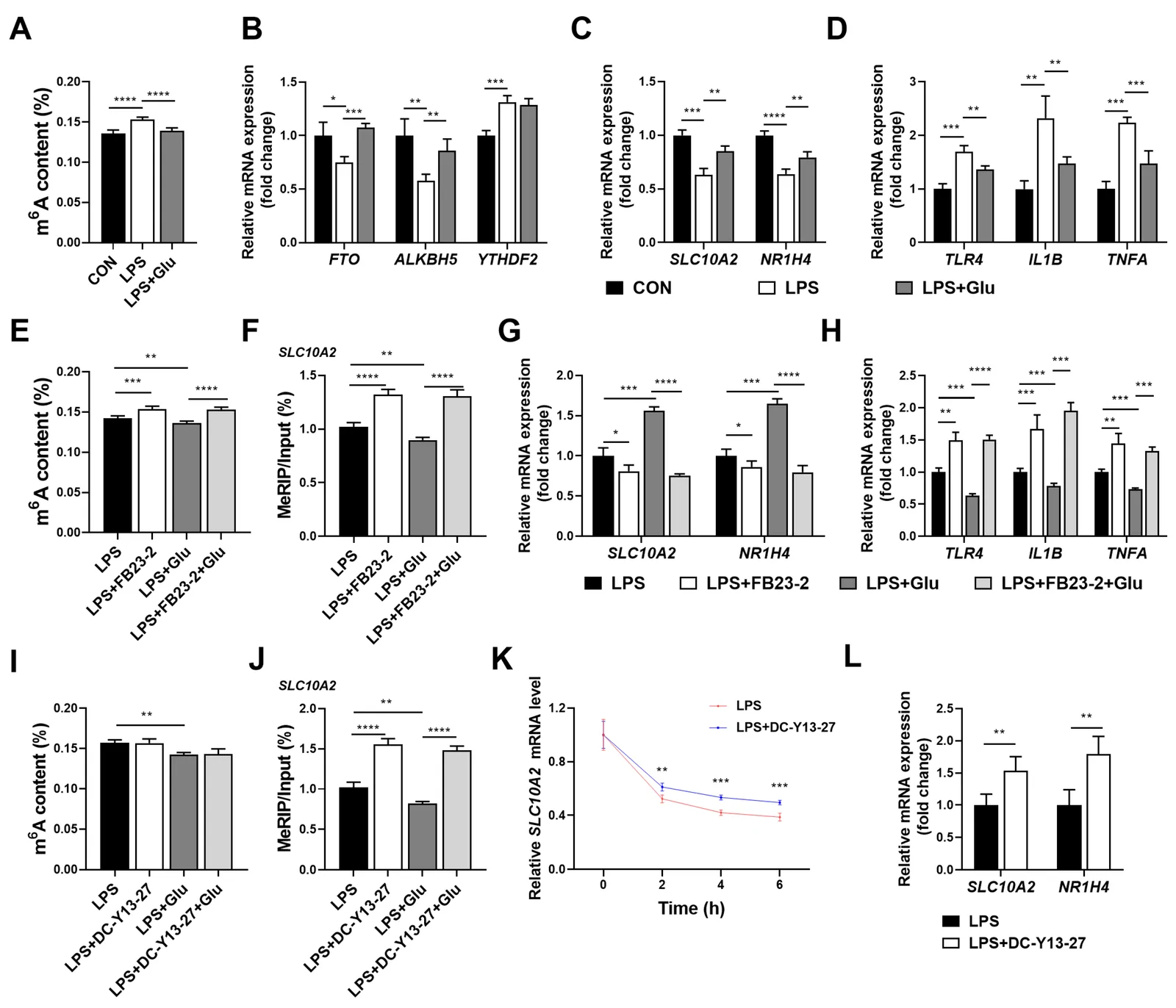
Glutamate regulates SLC10A2/ASBT expression in lipopolysaccharide (LPS)-stimulated IPEC-J2 cells through m^6^A demethylase- and YTHDF2-dependent mechanisms. (**A**–**D**) Effects of LPS and glutamate on m^6^A content and gene expression in IPEC-J2 cells from the control (CON), LPS challenge (LPS), and LPS challenge with glutamate supplementation (LPS + Glu) groups. (**A**) Total m^6^A content (n = 6 replicates). (**B**) Relative mRNA expression of FTO, ALKBH5, and YTHDF2 (n = 4 replicates). (**C**) Relative mRNA expression of SLC10A2 and NR1H4 (n = 4 replicates). (**D**) Relative mRNA expression of *TLR4*, *IL1B*, and *TNFA* (n = 4 replicates). (**E**–**H**) Effects of FTO inhibition (FB23-2, 10 μM) on glutamate-mediated changes in LPS-stimulated IPEC-J2 cells in the LPS challenge (LPS), LPS challenge with FB23-2 treatment (LPS + FB23-2), LPS challenge with glutamate supplementation (LPS + Glu), LPS challenge with FB23-2 treatment and glutamate supplementation (LPS + FB23-2 + Glu) groups. (**E**) Total m^6^A content (n = 6 replicates). (**F**) Relative mRNA expression of FTO, ALKBH5, and YTHDF2 (n = 4 replicates). (**G**) Relative mRNA expression of SLC10A2 and NR1H4 (n = 4 replicates). (**H**) Relative mRNA expression of *TLR4*, *IL1B*, and *TNFA* (n = 4 replicates). (**I**–**L**) Effects of YTHDF2 inhibition (DC-Y13-27, 5 μM) on SLC10A2 m^6^A modification and stability in LPS-stimulated IPEC-J2 cells in LPS challenge (LPS), LPS challenge with DC-Y13-27 treatment (LPS+ DC-Y13-27), LPS challenge with glutamate supplementation (LPS + Glu), and LPS challenge with DC-Y13-27 treatment and glutamate supplementation (LPS+ DC-Y13-27 + Glu) groups. (**I**) Total m^6^A content (n = 6 replicates). (**J**) MeRIP-qPCR analysis of m^6^A abundance on SLC10A2 mRNA (n = 4 replicates). (**K**) *SLC10A2* mRNA stability (n = 4 replicates). (**L**) Relative mRNA expression of *SLC10A2* (n = 4 replicates). Data were analyzed using two-tailed Student’s *t*-test and are represented as means ± SD. * *p* < 0.05, ** *p* < 0.01, *** *p* < 0.001, **** *p* < 0.0001. FTO, fat mass and obesity-associated protein; ALKBH5, AlkB homolog 5; YTHDF2, YTH N6-methyladenosine RNA-binding protein 2; ASBT, apical sodium-dependent bile acid transporter; TLR4, Toll-like receptor 4; MeRIP-qPCR, methylated RNA immunoprecipitation quantitative PCR; FB23-2, FTO-selective inhibitor; DC-Y13-27, YTHDF2-selective inhibitor.

Like the results in weaned piglets, LPS stimulation increased the total m^6^A content and *YTHDF2*, *TLR4*, *IL1B* and *TNFA* expression in pig intestinal epithelial cell line IPEC-J2 cells, while reducing the expression of *FTO*, *ALKBH5*, *SLC10A2* and *NR1H4*; adding glutamate to the culture medium reversed these effects (Figure 5A–D). Next, we investigated whether glutamate affects *SLC10A2* expression through m^6^A demethylase FTO and the reading protein YTHDF2. The addition of glutamate reduced m^6^A content, increased SLC10A2 and NR1H4 expression, and reduced TLR4, IL1B and TNFA expression in IPEC-J2 cells upon LPS stimulation; inhibiting FTO via its inhibitor FB23-2 eliminates the effects of glutamate addition (Figure 5E–H). The reader protein YTHDF2 promotes degradation of m^6^A-modified mRNA [28]; thus, we speculate that LPS-induced hyper-m^6^A methylation induces SLC10A2 degradation. As expected, inhibition of YTHDF2 by its inhibitor DC-Y13-27 in LPS and LPS + glutamate intervened IPEC-J2 cells did not affect overall m^6^A content, but increased m^6^A modification in SLC10A2 mRNA (Figure 5I,J). Moreover, inhibition of YTHDF2 increased mRNA stability of SLC10A2 in LPS-stimulated IPEC-J2 cells and increased its expression (Figure 5K,L). Taken together, glutamate reduces the m^6^A modification level of SLC10A2 by increasing m^6^A demethylase expression, thereby reducing YTHDF2-mediated SLC10A2 degradation.

### 3.5. Glutamate Supplementation Supports α-KG-Mediated m^6^A Demethylation in the Ileum

**Figure 6:**
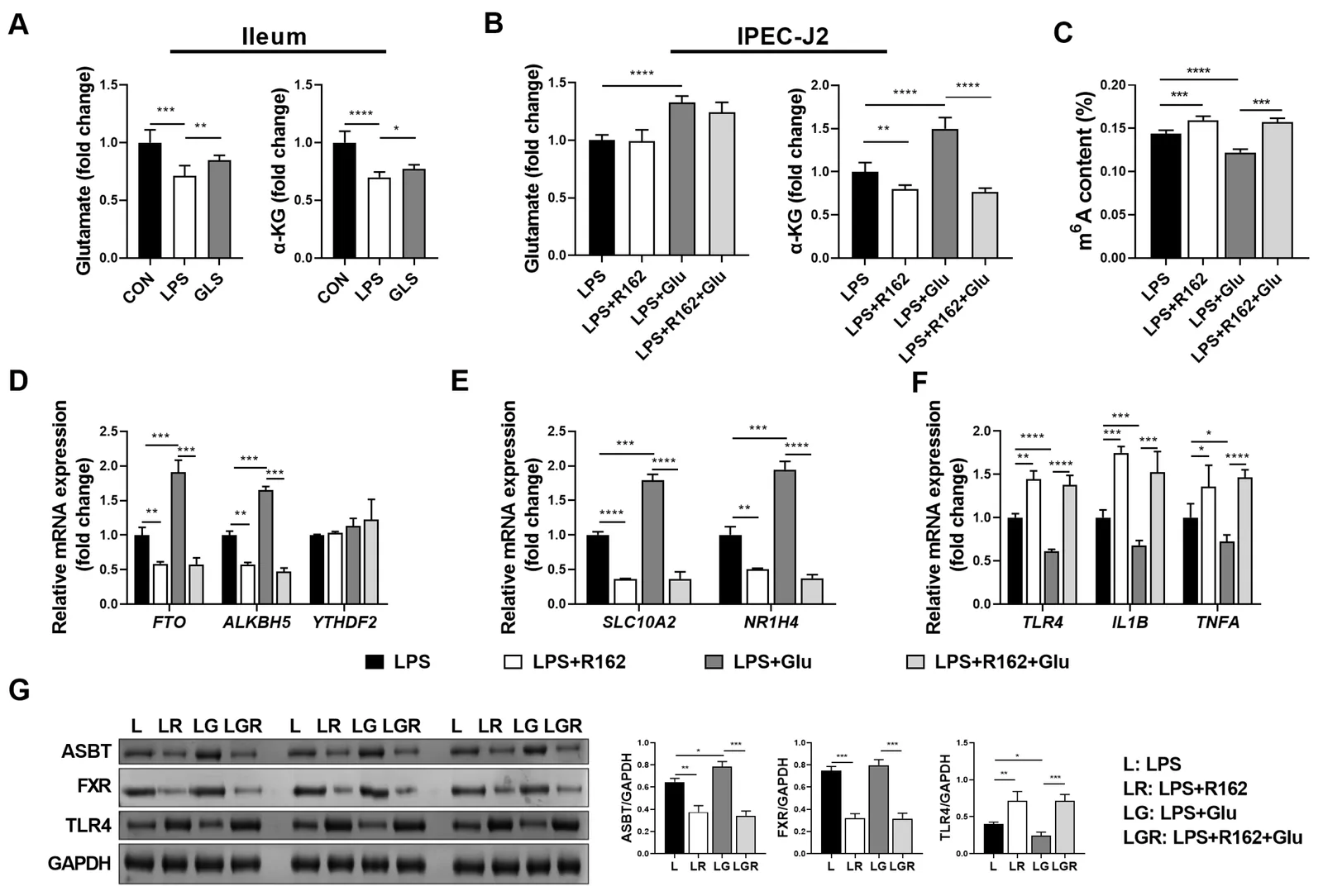
Glutamate supplementation maintains m^6^A demethylase and SLC10A2/FXR expression via conversion to α-KG. (**A**) Glutamate and α-ketoglutarate (α-KG) content in the piglet ileum (**A**) and IPEC-J2 cells from the LPS challenge (LPS), LPS challenge with R162 treatment (LPS + R162), LPS challenge with glutamate supplementation (LPS + Glu), and LPS treatment with R162 treatment and glutamate supplementation (LPS + R162 + Glu) groups (**B**) (n = 4 replicates). (**C**) The m^6^A content of IPEC-J2 cells treated as indicated (n = 6 replicates). (**D**–**F**). Relative mRNA expression level of FTO, ALKBH5, and YTHDF2, (**D**) SLC10A2 and NR1H4, and (**E**) TLR4, IL1B and TNFA (**F**) in IPEC-J2 cells treated as indicated (n = 4 replicates). (**G**). Protein abundance of ASBT, FXR and TLR4 (n = 3 replicates). Data were analyzed using two-tailed Student’s *t*-test and represented as means ± SD. * *p* < 0.05, ** *p* < 0.01, *** *p* < 0.001 and **** *p* < 0.0001. α-KG, α-ketoglutarate; FTO, fat mass and obesity-associated protein; ALKBH5, AlkB homolog 5; YTHDF2, YTH N6-methyladenosine RNA-binding protein 2; ASBT, apical sodium-dependent bile acid transporter; FXR, farnesoid X receptor; TLR4, Toll-like receptor 4; R162, glutamate dehydrogenase-selective inhibitor.

To further investigate how glutamate supplementation influences m^6^A modification in the ileum of LPS-challenged piglets, we then analyzed the levels glutamate and α-KG in the ileum. The results showed that the LPS group had lower levels of glutamate and α-KG in the ileum, while glutamate supplementation replenished the levels of glutamate and α-KG in the ileum (Figure 6). Glutamate is converted to α-KG by GDH. Next, we intervened with GDH (by its inhibitor R162) in IPEC-J2 to inhibit the conversion of glutamate to α-KG. Similar to the results in the piglet ileum, adding glutamate to IPEC-J2 cell culture medium increased the levels of intracellular glutamate and α-KG in LPS-stimulated cells, while R162 decreased α-KG levels in both LPS-stimulated cells and LPS + Glu treatment cells (Figure 6B). The addition of glutamate also reduced the intracellular m^6^A levels and increased the expression of *FTO* and *ALKBH5* expression in IPEC-J2 cells stimulated by LPS, while R162 intervention eliminated this function (Figure 6C,D). As expected, the addition of glutamate also increased the expression of *SLC10A2* and *NR1H4* in LPS-stimulated IPEC-J2 cells, thereby reducing the mRNA expression of TLR4, IL1B and TNFA, as well as protein expression levels of ASBT, FXR and TLR4, while GDH inhibition directly eliminated the replenishment effect of glutamate (Figure 6E–G). In general, glutamate supports intracellular α-KG levels and upregulates m^6^A demethylase expression.

## 4. Discussion

Glutamate is well established as a primary energy substrate for the intestinal epithelium of weaned piglets and participates in the functional regulation of intestinal immune cells, including macrophages. Nevertheless, the precise mechanism by which glutamate modulates intestinal immune homeostasis in weaned piglets and its potential association with epigenetic modification remains poorly defined. In this study, we found that dietary glutamate supplementation ameliorates LPS-induced ileal inflammatory injury and restores intestinal morphological integrity in weaned piglets. However, several links in this model remain inferential. For example, we did not directly measure FTO or ALKBH5 enzymatic activity, nor did we perform site-directed mutagenesis of SLC10A2 m^6^A sites to definitively establish causality between m^6^A modification and transcript stability. Additionally, the direct activation of FXR by bile acids was not functionally assessed in this study. These mechanistic links should be interpreted as hypotheses supported by correlative and pharmacological evidence rather than definitively proven pathways.

This study has several limitations that should be acknowledged. The animal experiment used an acute LPS challenge model, which induces a rapid systemic inflammatory response but does not fully recapitulate the complex, multifactorial nature of naturally occurring weaning-associated intestinal inflammation or post-weaning diarrhea. Therefore, the findings should be interpreted within the context of acute endotoxemia rather than as a direct model of weaning stress. Besides this, the study focused on the ileum; whether similar mechanisms operate in other intestinal segments or in other immune cell types (e.g., macrophages) remains to be determined. Future studies should address these limitations using genetic manipulation, direct FXR activity assays, and more comprehensive sampling across intestinal segments and cell types.

Dietary amino acid intervention is recognized as an effective strategy to modulate intestinal immune function in piglets [8,9]. However, the molecular mechanism of amino acid regulation of immune cell function, especially the epigenetic pathway, has not been fully elucidated. Specifically, the deletion of GAT2, a transporter responsible for mediating the transport of glutamate metabolite γ-aminobutyric acid (GABA), elevates DNA methylation levels at the KID3 gene promoter and enhances oxidative phosphorylation in macrophages [29]. Additionally, GABA treatment reduces histone methylation abundance in macrophages, thereby restricting the release of pro-inflammatory IL-1β [30], indicating a deep interaction between amino acid metabolism and epigenetic modification networks. However, the relationship between amino acid metabolism and epigenetic modifications at the mRNA level is not yet clear. We found that LPS challenge leads a decrease in intracellular glutamate/α-KG thereby inducing hyper-m^6^A modification of ileal cells. Glutamate provides α-KG to reduce the m^6^A modification level of bile acid reabsorption gene *SLC10A2*, thereby increasing its expression and ultimately activating FXR to alleviate intestinal inflammation. Consistent with our results, emerging evidence has verified that m^6^A epigenetic modification acts as a pivotal molecular bridge linking cellular metabolic reprogramming and intestinal immune regulation. Dysregulated m^6^A deposition profoundly alters the stability, splicing and translation efficiency of inflammation- and metabolism-related mRNAs, thereby participating in the occurrence and progression of intestinal mucosal injury [31,32]. Specifically, metabolic stress-induced α-KG deficiency is widely recognized as a core driver of hyper-m^6^A modification [33,34], while glutamate supplementation effectively replenishes intracellular α-KG reservoirs to reverse aberrant m^6^A methylation patterns, stabilize target gene expression and restore metabolic homeostasis. In line with previous epigenetic studies on amino acids, glutamate-derived metabolites also regulate DNA and histone methylation to reshape mast cell functions [7], further confirming the universal regulatory role of amino acid metabolism in multiple epigenetic modification networks.

The current study highlights the close interaction between intestinal bile acid metabolism, epigenetic modification and innate immunity. Intestinal BA pool homeostasis is not only essential for nutrient digestion and absorption but also serves as a critical upstream determinant of the intestinal immune microenvironment [35,36]. Aberrant m^6^A modification induced by amino acid metabolic disorder disrupts bile acid reabsorption, which in turn locks the intestinal epithelium into a persistent inflammatory state, forming a vicious cycle of “metabolic disorder–epigenetic abnormality–intestinal inflammation”. Targeted restoration of glutamate/α-KG metabolism effectively breaks this pathological cycle via m^6^A demethylation-dependent stabilization of SLC10A2 and FXR signal activation. Considering that, except for NF-κB signaling, pathways such as STAT, mTOR and MAPK are also involved in regulating FXR activity [37,38,39]. Future studies should further explore the crosstalk between the m^6^A-FXR axis and classic metabolic and inflammatory signaling pathways, as well as the regulatory effect of amino acid nutrition on intestinal macrophage polarization and mucosal autophagy via epigenetic modification.

## 5. Conclusions

In summary, the present work provides evidence that dietary glutamate supplementation is associated with alleviation of LPS-induced intestinal inflammatory injury in weaned piglets. The data suggest that glutamate-derived α-KG may support the expression of m^6^A demethylases FTO and ALKBH5, which is associated with reduced m^6^A modification of SLC10A2 mRNA and limited YTHDF2-mediated transcript degradation, potentially restoring ileal bile acid reabsorption and FXR signaling to restrain overactivated TLR4/NF-κB inflammatory responses. These findings support a glutamate–α-KG–m^6^A–SLC10A2–FXR axis that may contribute to intestinal protection under acute inflammatory stress, offering a potential epigenetic target for nutritional intervention in weaned piglets. Further studies using genetic and functional approaches are warranted to establish the causal relationships among these mechanistic links.

## Data Availability

The data that support the findings of this study are available from the corresponding author upon reasonable request.

## Supplementary Materials

The following supporting information can be downloaded at https://www.mdpi.com/article/10.3390/ani16182854/s1. Table S1: Composition and nutrient levels of the basal diet; Table S2: Key resources table; Table S3: Primer sequences used in quantitative real-time PCR assays; Table S4: Primer sequence for MeRIP-PCR.
