# Supplementary material for: Dietary Supplementation of Glutamate Alleviates LPS-Induced Intestinal Injury Associated with m6A Modification of SLC10A2 in Weaned Piglets
Source: Animals (Basel). 2026 Sep 10;16(18):2854. doi: 10.3390/ani16182854 (PMC13603767; doi:10.3390/ani16182854)
Supplement: Supplementary file 1 [file animals-16-02854-s001.zip › animals-4528714-supplementary.pdf]

Table S1. Composition and nutrient levels of the basal die.

Items	Content
Ingredients	%
Maize	65.8
Soybean meal	24
Soybean oil	2.5
Fish meal	1.5
Limestone	0.85
$CaHPO_4$	1
Salt	0.35
Premix[a]	4
Nutrient	
Digestive energy (MJ/kg)[b]	1525
Protein (%)[b]	18.5
Calcium (%)[b]	0.70
Phosphorus (%)[b]	0.55
Lysine (%)[b]	1.32
Crude fiber (%)[b]	3.25

[a] Supplied per kilogram of diet: vitamin A, 2200 IU; vitamin D-3, 220 IU; vitamin E, 16 IU; vitamin K-3, 0.5 mg; biotin, 0.05 mg; folic acid, 0.3 mg; pantothenic acid, 10 mg; niacin, 15 mg; vitamin B2, 3.6 mg; vitamin Bl, 1.0 mg; vitamin B6, 1.5 mg; Cu, 6 mg; Fe, 100 mg; Zn 110 mg; Mn, 4 mg; Se, 0.3 mg; I, 0.14 mg.

[b] Calculated value.

Table S2. KEY RESOURCES TABLE.

REAGENT or RESOURCE	SOURCE	IDENTIFIER
**Antibodies**		
ZO-1	Proteintech	21773-1-AP
OCLN	Proteintech	66378-1-Ig
CLDN	Proteintech	28674-1-AP
GAPDH	Proteintech	60004-1-Ig
TLR4	Proteintech	66350-1-Ig
p-p65	CST	3033T
p65	CST	8242T
ASBT	Proteintech	25245-1-AP
OST	Proteintech	31330-1-AP
FXR	CST	72105T
FTO	Proteintech	68111-1-Ig
ALKBH5	Proteintech	16837-1-AP
YTHDF2	Proteintech	24744-1-AP
**Chemicals**		
FB23-2	MCE	HY-127103
DC-Y13-27	MCE	HY-154919
R162	MCE	HY-103096
**Software**		
Image J	NIH	
Graphpad Prism 8	Graph Pad Software	
QuantStudioTM Real-Time PCR V1.7.2	QuantStudio™ Real-Time PCR Software	

Table S3. Primer sequences used in quantitative real-time PCR assays.

Gene	Accession no.	Primer, 5′–3′
β-actin	XM_003124280.4	CGGCATCCACGAAACTACCT
		ACTCCTGCTTGCTGATCCAC
METTL3	XM_003128580.5	TGAGGCTCCTGGAAGCAAAG
		TCTGTCAGGGTCCCATAGGG
METTL14	XM_003129231.6	GTGGTTCTGGGGAGGGATTG
		GAAGTCCCCGTCTGTGCTAC
FTO	KM232950.1	CCCCAGAAAATGCCGTACCT
		ACCAGGGGTCTCTATGTCCC
ALKBH5	XM_021067995.1	CGTGTCCGTGTCCTTCTTCA
		AGGATGATGACAGCTCTGCG
YTHDF1	MN606020.1	GCACCGCTCCATCAAGTACT
		GCTGAAGAGCAGGTAGACGG
YTHDF2	XM_005665152.3	CCAAGGGATGGCAGCACTAA
		TTTGCCACAGGACCCTTGTT
YTHDF3	MN606021.1	GAGCAAGGCATGACTGGACT
		CTGGGGGCACACTATTGGTT
IL1B	NM_001302388.2	GCTGATGGCCCCAAAGAGAT
		TGCCACAATCACAGACACCA
TNFA	NM_214022.1	TCCAATGGCAGAGTGGGTATG
		AGCTGGTTGTCTTTCAGCTTCAC
TLR4	GQ503242.1	TCAGTTCTCACCTTCCTCCTG
		GTTCATTCCTCACCCAGTCTTC
SLC51A	NM_001244266.1	GTTTCTCATCCCTGACGGCA
		GAGGAAAAGGGAGGCGAACA
SLC51B	XM_003121716	GGCTGTGGTGGTCGTGATAA
		TCTCAGATTCCCGTGGGTCT
SLC10A2	NM_001244463	TTCCTTTGGCCGGTTACTCC
		TGCCCTCTGGAAATTCCACC
NR1H4	KF534517	TGAGCTTTGTGTCGTTTGCG
		GATCTGCATGCTGCTTCACG

Table S4. Primer sequence for MeRIP-PCR.

Gene	Accession no.	Primer, 5′–3′
SLC10A2	NM_001244463	GCAGAAAACCCAGGGATGGA
		TTCCACGTTACACCCCATGG
